# Use of Piezoelectric Immunosensors for Detection of Interferon-Gamma Interaction with Specific Antibodies in the Presence of Released-Active Forms of Antibodies to Interferon-Gamma

**DOI:** 10.3390/s16010096

**Published:** 2016-01-20

**Authors:** Elena Don, Olga Farafonova, Suzanna Pokhil, Darya Barykina, Marina Nikiforova, Darya Shulga, Alena Borshcheva, Sergey Tarasov, Tatyana Ermolaeva, Oleg Epstein

**Affiliations:** 1 OOO “NPF “MATERIA MEDICA HOLDING”, 3rd Samotyochny per., 9, 127473 Moscow, Russia; PokhilSE@materiamedica.ru (S.P.); LubshinaDV@materiamedica.ru (D.B.); MashkinaMV@materiamedica.ru (M.N.); ShulgaDP@materiamedica.ru (D.S.); BorshchevaAA@materiamedica.ru (A.B.); TarasovSA@materiamedica.ru (S.T.); Nauka@materiamedica.ru (O.E.); 2 Lipetsk State Technical University, Moskovskaya ul, 30, 398006 Lipetsk, Russia; voronezkaya@gmail.com (O.F.); ermolaeva@stu.lipetsk.ru (T.E.)

**Keywords:** released-active forms of antibodies, piezoelectric immunosensors, interferon-gamma

## Abstract

In preliminary ELISA studies where released-active forms (RAF) of antibodies (Abs) to interferon-gamma (IFNg) were added to the antigen-antibody system, a statistically significant difference in absorbance signals obtained in their presence in comparison to placebo was observed. A piezoelectric immunosensor assay was developed to support these data and investigate the effects of RAF Abs to IFNg on the specific interaction between Abs to IFNg and IFNg. The experimental conditions were designed and optimal electrode coating, detection circumstances and suitable chaotropic agents for electrode regeneration were selected. The developed technique was found to provide high repeatability, intermediate precision and specificity. The difference between the analytical signals of RAF Ab samples and those of the placebo was up to 50.8%, whereas the difference between non-specific controls and the placebo was within 5%–6%. Thus, the piezoelectric immunosensor as well as ELISA has the potential to be used for detecting the effects of RAF Abs to IFNg on the antigen-antibody interaction, which might be the result of RAF’s ability to modify the affinity of IFNg to specific/related Abs.

## 1. Introduction

Drug products containing antibodies (Abs) or their derivatives as active ingredients have been successfully used for the prevention and treatment of severe and socially significant diseases, such as cancer, multiple sclerosis, influenza, acute respiratory tract viral infections, diabetes, *etc.* [1,2]. The principle interaction underlying therapeutic effects of such medicines is the antigen-antibody (Ag-Abs) interaction, the identification and quantification of which is therefore an important target for immunoassays [3,4]. To date, a variety of corresponding assays have been available, differing in the method of detecting this interaction: (1) direct detection where the Ag-Abs complex is identified either visually or by simple optical devices [5,6]; (2) detection based on the passive agglutination reaction, *i.e.*, adhesion of particles with Ag or Abs bound to their surfaces [7,8]; (3) detection relying on the use of various labels [9,10,11,12,13,14]; (4) detection techniques employing rapid, portable, high-sensitivity sensors. The latter include immunochromatographic systems [15,16], immunochips [17], or immunosensors [14,18], which represent a fast-developing approach in laboratory analysis using various types of biologically sensitive elements to furnish information about the Ag-Abs interaction in the form of electrical signals [19]. The important distinction of immunosensor assays is the method of signal transduction which may employ either an electrochemical or an optical transducer [20]. In electrochemical transducers, an electrochemical signal is measured, the magnitude of which changes following the formation of the Ag-Abs complex [21,22]. In the case of optical transducers, Ag-Abs quantification is based on the generation of light signals (color change or fluorescence) or changes in the optical properties of the environment [23]. Among major advantages of immunosensors are the rapidity of analysis, convenience of use, possibility of quantification with subsequent mathematical processing, portability of the test devices and high sensitivity [19].

One of the most promising biosensor options is piezoelectric immunosensors (PI) designed on the basis of quartz crystal resonators (QCR), which are intended for the detection of bacteria, identification of biological nanoparticles or registration of immune interactions [24]. PI enables direct detection of biochemical interactions between receptor molecules without the need for additional labeling (fluorescent, enzymatic, *etc.*) which favorably distinguishes them from similar devices. This type of immunosensor is characterized by fast response and ease of operation, integrability into multisensory systems or automatic data acquisition systems [25]. The unparalleled feature of PI is that they compromise high sensitivity ensured by the use of QCR as a transducer element and selectivity, which is determined by the specificity of receptor molecules employed [19]. The principle behind PI operation is the detection of frequency variations of the electrical signal following Ag-Abs complex formation, resulting in an increase of mass sensed by the quartz plate of the resonator or cantilever. The specific biolayer in a QCR provides highly selective identification of homologous antigens in a complex mixture with no need for additional procedures involving other reagents.

This work is aimed at demonstrating the possibility of using PI as an efficient tool to tackle novel tasks, in particular those associated with analyzing the effects of released-active forms of Abs to interferon-gamma (RAF Abs to IFNg) on immune interactions at a molecular level.

The released-activity (RA) phenomenon was discovered during studies of a processing technique involving repeated serial steps of decreasing the concentration of the initial substance in combination with external treatment at each step [26,27]. Dilutions prepared using this technique were found to share a common feature—the ability to directly modify the initial substance, alter its spatial structure and, consequently, its physicochemical and biological properties. RAF-based drug products are referred to as “released-active”, with their efficacy and safety proven by both non-clinical and clinical studies [28,29,30,31,32,33,34,35,36,37,38,39]. We also previously demonstrated the possibility of using ELISA tests for examining the effects of both antibody and non-antibody RAFs on the Ag-Abs interaction [40,41]. Development studies of RAF Abs to IFNg using ELISA showed that the binding constant of commercial Abs to IFNg was changed in the presence of RAF Abs to IFNg, indicating that RAF Abs to IFNg can influence steric interaction [40]. Because the characteristics of PI are largely dependent on the concentration and steric availability of biomolecules immobilized onto the electrode surface, we suggested that this approach could be used for identification tests and assays of RAF Abs. For this purpose, we selected a PI technique employing high-sensitivity sensors in order to evaluate the effects of RAF Abs to IFNg on the Ag-Abs interaction. This provided us with the data elucidating the influence of RAF Abs on Abs’ affinity to IFNg; the findings are presented in this paper.

## 2. Experimental Section

### 2.1. Experimental Design

For the study, a piezoelectric immunosensor was used to detect the Abs-Ag interaction between Abs to IFNg and IFNg in the presence of RAF Abs to IFNg or control samples. As a physical transducer, a high-frequency piezoelectric AT-cut quartz crystal resonator was used, operating on the Bulk Acoustic Wave (BAW) principle where the resonance occurs across the entire mass of the crystal. The device is operated at a frequency of 10 MHz ± 1 Hz and based on gold nanoparticles assembled onto 8 mm diameter electrodes and providing active sites for the immobilization of Abs molecules (ZAO “ETHNA”, Moscow, Russia).

Variation in oscillation frequency of the quartz crystal resonator (Δf) is the analytical signal in piezoelectric immunosensors and this parameter is dependent on increases or decreases in the mass of bioreceptor. Such mass changes arise from the formation or disintegration of immune complexes on the sensor surface [42]. Analysis of liquids with the use of piezoelectric sensors can be carried out both in a static and a flowing mode. In the first case (the procedure being known as “dip and dry”), increase of the receptor layer mass is measured before and after contact of the sensor with the analyzed liquid sample and further air-drying to constant mass, whereas in the second case (flowing mode) signal from the sensor is measured continuously [19]. The test samples were analyzed in the flowing mode. Using sensor as a detector for flow-injection analysis makes it possible to raise the speed of determination and also provides the opportunity of observing immunochemical reactions in real time. The analysis employed dosage unit samples, power unit, 15–20 µL flow cell providing contact with the analyzed sample only from one end of the sensor, peristaltic pump (KNAUER, Berlin, Germany), drive circuit (TTL based on IC74LS320) transforming mass changes of the bioreceptor coating into frequency fluctuations, frequency-measuring device DiScope (NPP “Bafika”, Moscow, Russia) and personal computer.

The tests were performed using 11-mercapto-1-undecanol surface coating with the electrode oscillation frequency recorded every 10 s. Liquid samples were utilized without prior preparation. Lactose and tablet samples in the amount of 30 mg were dissolved in 1 mL of distilled water at room temperature. The tablets were initially grounded using a porcelain mortar and pestle and, after the dissolution, heated at 37 °C for 45 min followed by centrifugation at 1000 rpm for 10 min. Samples of each type were combined with IFNg in 4:1 (sample:IFNg) ratio and incubated at 37 °C for 60 min followed by centrifugation at 8000 rpm for 10 min and measurements of analytical signals of the samples. The results were obtained by processing the analytical signals recorded over 10-day experimentation (five readings by each of the three operators engaged, a total of 150 readings).

### 2.2. Test Samples

RAF Abs to IFNg were supplied for testing in the form of ready-to-use solutions, tablets, and lactose powder by OOO “NPF “MATERIA MEDICA HOLDING” (Moscow, Russia). Affinity purified polyclonal rabbit antibodies to recombinant human IFNg were manufactured by Angel Biotechnology Holdings plc (Edinburgh, UK) as the starting material for commercial production of an oral therapeutic Anaferon for Children in accordance with the current European Union GMP requirements for starting materials [43]. RAF Abs to IFNg were obtained using routine methods described in the European Pharmacopoeia [44]. Briefly, antibodies to IFNg (2.5 mg/mL) were mixed with a solvent (ethanol-water solution) and shaken for 1 min to produce C1 dilution (first centesimal dilution). All subsequent dilutions comprised one part of the previous dilution and 99 parts of solvent (ethanol-water solution for intermediate dilutions and distilled water for the preparation of final dilution), with succession between the dilution steps. Thus, RAF of Abs to IFNg contain released-active dilutions of antibodies, which were diluted up to receiving the mixture of final dilutions C12, C30 and C50. The solutions were prepared in sterile conditions protected from direct intense light and stored at room temperature. All dilutions were prepared in glass vials. For lactose sample preparation, lactose monohydrate powder was saturated with RAF Abs to IFNg on a 96 L Pilotlab fluid bed apparatus (Hüttlin GmbH, Schopfheim, Germany). In case of tablet form, each tablet was made from such lactose powders saturated with RAF Abs to IFNg and auxiliary components by compression molding.

In the case of placebo, solvent (buffer solution which is equivalent to the vehicle for Abs to IFNg) was used to prepare RAF of buffer (RAF B) instead of Abs to IFNg following the method described above. This solution of RAF B was used to prepare the lactose and tablet placebo samples. RAFs of non-specific controls were prepared similarly to RAF Abs to IFNg but using Abs to tumor necrosis factor alpha (TNFa) (Sigma-Aldrich, St. Louis, MO, USA) and diclofenac sodium salt (Sigma-Aldrich, St. Louis, MO, USA) as respective starting materials instead of Abs to IFNg. RAF Abs to IFNg. Placebo and non-specific controls were coded by the manufacturer and examined blind during the study.

### 2.3. Materials

Standard IFNg solutions (100 µg/mL) were prepared by dissolving IFNg (Abcam, Cambridge, UK) in 1 mL of bidistilled water. The antibody component was represented by affinity purified polyclonal rabbit Abs to human IFNg (U-CyTech Bioscience B.V., Utrecht, The Netherlands) at concentration of 9.6 ng/mL. Working solutions of Abs to IFNg were prepared without performing dilution or by diluting them two, four and eight times in phosphate-buffered saline (PBS) 7.2 containing (g/L): NaCl (8.0), KCl (0.2); Na_2_HPO_4_∙12H_2_O (2.9), and KH_2_PO_4_ (0.2). The following organic solvents were used: dimethylformamide (DMFA), acetone (Esteve Quimica, Celrà, Spain), methanol, ethanol, chloroform (“Reachem”, Moscow, Russia). The non-organic reagents included: hydrochloric acid, sodium chloride, sodium hydrophosphate, sodium tetraborate, sodium azide, potassium chloride, potassium dihydrophosphate, potassium thiocyanate and ammonium sulfate (“Reachem”, Moscow, Russia). The cross-linking reagents used were as follows: glutaraldehyde (GA), 1-ethyl-3-(3-dimethylaminopropyl) carbodiimide (EDAC), N,N′-dicyclohexylcarbodiimide (DCC), N-hydroxysuccinimide (NHS), cystamine (Reanal, Budapest, Hungary). The following reagents were used as regeneration agents: potassium rhodanide, hydroxylamine hydrochloride, carbamide, phenol, diethylamine hydrochloride and magnesium chloride in different concentrations, and bidistilled water (“Reachem”, Moscow, Russia). The compounds utilized to prepare a thin-layer substrate included: 2-amino-3-mercaptopropionic acid (MPA), 11-mercaptoundecanol (MU), 2-mercaptoethylamine (Sigma-Aldrich, St Louis, MO, USA), (2-oxy-3-mercaptoethyl)-3-benzoylpyrimidine (OMBP), 2-*N*-4-mercapto-6-phenyl-1,3,5-triazine (MPT), 2-amino-5-mercapto-1,3,4–triazole (AMT) (Lancaster Synthesis, Ward Hill, MA, USA), γ-aminopropyltriethoxysilane (APTS) (Reanal, Budapest, Hungary), calix[4]arene (Fluka, Buchs, Switzerland).

### 2.4. Selection of Receptor Layer Preparation Conditions

The electrode was coated with the substrate material and Abs to IFNg (solution concentrations 9.6 ng/mL, 4.8 ng/mL, 2.4 ng/mL, 1.2 ng/mL) were adsorbed. After depositing IFNg solution onto the substrate, the signal was recorded. Five methods of Abs adsorption were chosen: (1) adsorption of Abs to IFNg immobilized onto APTS-coated substrate with EDAC and GA used as cross-linkers. For a siloxane layer, 0.8 μL of APTS was spot-coated onto the surface with a microsyringe and oven0-dried at 80 °C for 20 min followed by the addition of 5 μL of GA (2.5% solution in bidistilled water) or EDAC (2% solution in acetonitrile). Alternative method: (2) gold electrodes were coated with 1 μL of 2-amino-3-mercaptopropionic acid (0.1% solution in ethanol) and incubated at 22–23 °C for 8 h. Thereafter the electrode surface was rinsed with ethanol and 5 μL of EDAC (2% solution in acetonitrile) or NHS (2 mM solution in ethanol) was added. Following 20-min incubation at 22–23 °C and further 12-h incubation at +4 °C, the treated electrode was rinsed with PBS. Another Abs adsorption technique (3) involved coating the sensor surface with 5 μL of 11-mercapto-1-undecanol and incubation at room temperature for 30 min. Following this, 5 μL of GA (2.5% solution in bidistilled water) was deposited with subsequent incubation at 4 °C for 12 h and PBS rinse. For the next method (4), 5 μL of 11-mercapto-1-undecanol (MU) and 5 μl of (2-oxy-3-mercaptoethyl)-3-benzoylpyrimidine (OMBP) (or 2-*N*-4-mercapto-6-phenyl-1,3,5-triazine (MPT) or 2-amino-5-mercapto-1,3,4-triazole (AMT)) were successively coated onto a freshly cleaned electrode surface and incubated in a drying oven at +80 °C for 1 h. Thereafter, 5 μL of freshly prepared 5% glutaraldehyde was added and after 15–20 min the sensor was carefully rinsed with buffer. Finally, for method (5), the sensor surface was coated with 8 μL of 2-mercaptoethylamine and incubated at room temperature for 30 min. Further, the surface was activated by depositing 5 μL of GA (2.5% solution in bidistilled water) with subsequent incubation at 4 °C for 12 h and buffer rinse or by 7 μL of 2% calix[4]arene followed by 12-h incubation at 4 °C and buffer rinse. Analytical signal measurements were performed in the flow-injection mode at flow rate of 60 μL/min for PBS.

Regeneration of the receptor layer was carried out so that it could be reused following changes of the analytical signal. For this, 100 μL of chaotropic agent was passed over the sensor surface, resulting in dissociation of the heterogeneous IFNg-Abs affinity complex and allowing avoiding destruction of the substrate. Different solution concentrations of potassium rhodanide, hydroxylamine hydrochloride, urea, phenol, diethylamine hydrochloride, and magnesium chloride at different concentrations as well as bidistilled water were tested as regeneration agents. Stability and duration of PI operation were evaluated by the number N of measurement runs where the analytical signal was not decreased by more than 5%.

### 2.5. Statistical Analysis

Data processing and statistical analysis were performed using SAS-9.3 statistical software. Data analysis for the main part of the experiment was carried out using a one-way analysis of variance (ANOVA). Test repeatability was assessed using Gauge Repeatability and Reproducibility Analysis (GRR). Testing of a series of chaotropic agents for potential use as regeneration agents did not require any statistical analysis. The data are presented as group means ± standard deviations unless indicated otherwise. *p*-values < 0.05 were regarded as statistically significant.

## 3. Results and Discussion

### 3.1. Development of the Assay

The initial experimental stage included studies to define the conditions of the receptor layer formation that provide enhanced analytical signals. This stage included the selection of coating treatment, regeneration agent, and “Sample:Antigen” ratio.

Five different coating treatments were examined (see Experimental Section), showing that the optimal properties, considering the cost of coating and magnitude of the analytical signal, were ensured by the treatment employing 11-mercapto-1-undecanol activated by glutaraldehyde. In the studies examining different Abs concentrations, the maximum signal magnitude was observed for 2.4 ng/mL Abs samples. Differences in the analytical signal obtained with different coating treatments are presented in Supplementary Table S1.

To allow repeated use of the receptor layer, regeneration with a chaotropic solution was performed following analytical signal measurements which resulted in the dissociation of the heterogeneous IFNg-Abs affinity complex without substrate destruction. The solutions of 0.003 M potassium rhodanide, 0.06 M diethylamine, and 0.1 M hydroxylamine hydrochloride were demonstrated to be the most effective regeneration agents ensuring fast regeneration of the sensor and its durability (Supplementary Table S2).

For quantification of the effects of test samples on the specific IFNg-Abs interaction, it was necessary to define the proper “sample:IFNg” ratio. The greatest differences were obtained for 2:1 and 4:1 “sample:IFNg” ratios which were further employed in the tests. Repeatability studies involved testing of both ratios, with the best results obtained for 4:1 (results are not presented due to data abundance). In this way, the conditions for PI measurements were selected based on the data provided by the studies described above.

### 3.2. Optimization of the Assay

**Figure 1:**
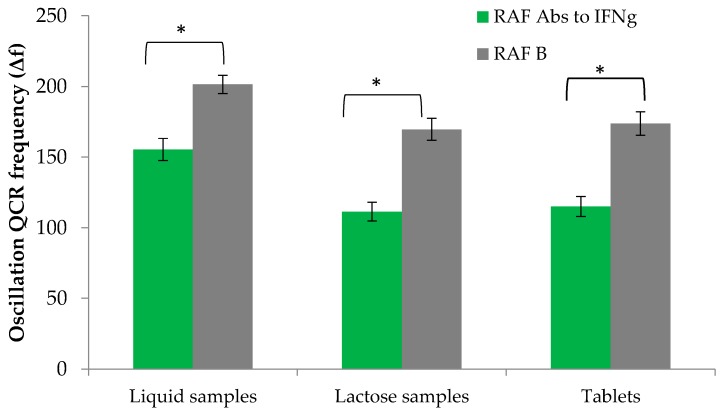
Intermediate precision: analytical signals of test samples and differences of RAF Abs to IFNg (**green**) *vs.* control (**grey**) as measured by three operators over a 10-day period (*n* = 150. *p* = 0.95). The full data is presented in the Supplementary Table S4. * means Pr > F is <0.0001.

The main experimental part was aimed at testing the RAF of the samples using the developed test procedure under the defined conditions. Since the analytical signal was shown to diminish in the presence of RAF Abs to IFNg, more reliable results of Abs to human IFNg detection were expected with regard to indirect measurements by comparing the analytical signal of the sensor obtained with IFNg solutions containing or not containing the RAF of specific Abs. Considering the available ELISA [40], we suggested that the effects of RAF Abs to IFNg could be evaluated using pre-incubation with one of the components of the Ag-Abs system for compounds containing RAF Abs to IFNg. However, variations of the analytical signal measured with PI may occur not only as a result of specific IFNg-Abs interactions, but also due to dilution or ionic strength changes of the analyzed solution, or non-specific interactions of secondary components with IFNg and the receptor layer. Hence, the presence of RAF Abs to IFNg in a sample can be demonstrated with higher reliability when differential evaluation of the signal is undertaken, with account for the noise signal, by examining the difference between the signal obtained in the presence of RAF Abs to IFNg and that of the placebo. The evaluation described was carried out, showing that the analytical signal was decreased following pre-incubation of Abs to IFNg with RAF Abs to IFNg which indicated attenuated affinity between IFNg and specific Abs in the presence of RAF Abs to IFNg. For instance, the decrease of the analytical signal was greater for RAF Abs to IFNg as compared with the placebo: the differences in signal magnitude were 19.4% (*p* ≤ 0.0001) for tablets, 51% (*p* ≤ 0.0001) for liquid samples and 34.3% for lactose powders (Figure 1).

For repeatability (intra-assay precision) evaluation, mean analytical signal values from Supplementary Table S3 were used with the ratio “sample:IFNg” of 4:1. The data is presented as ΔF = Δf (control) − Δf (test sample), where Δf is an oscillation frequency of the sensor following Abs-IFNg binding (Hz); s is a standard deviation; and δ is a confidential interval.

The s values, as indicated in Table 1, are the repeatability parameter and determine the precision of net signal measurements performed over a short time period during Abs to IFNg detection in liquid samples, lactose samples and tablets. The obtained s values reflect a high degree of repeatability.

**Table 1:** Repeatability (intra-assay precision): evaluation of net signals obtained for target samples *vs.* control.

Sample	Δf Mean ± δ	S	ΔF ± δ	s
RAF Abs to IFNg	124 ± 6	5	41 ± 2	1.6
RAF B	165 ± 4	3		
Lactose powders containing RAF Abs to IFNg	109 ± 13	11	38 ± 7	5.6
Placebo lactose powders	147 ± 6	5		
Tablets containing RAF Abs to IFNg	114 ± 7	6	83 ± 7	5.6
Placebo tablets	190 ± 7	6		

The evaluation of intermediate precision was performed in terms of a single 10-day study examining analytical results obtained by different operators (a total of 150 measurements). Relative standard deviation was approximately 4.94% (4.14% for water solutions, 5.25% for lactose powders, 5.44% for tablets), pointing to high closeness of results obtained by different operators on different days to the mean value and, therefore, high procedure reproducibility (Figure 1, Supplementary Table S4) [45].

**Figure 2:**
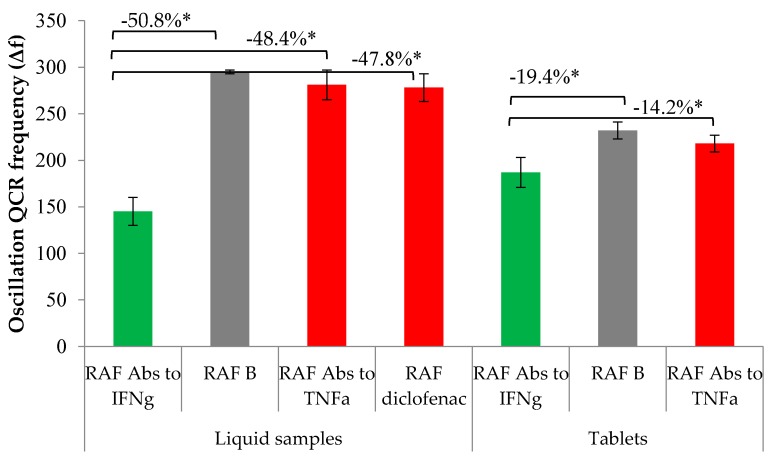
Specificity: analytical signals of test samples (**green**), controls (**grey**) and non-specific controls (**red**).

Specificity of the analytical procedure developed was tested using the following non-specific controls: RAF Abs to TNFa, RAF diclofenac—in the form of tablets and solutions. As non-specific lactose controls were unavailable, these were not used in testing. As opposed to RAF Abs to IFNg, the differences in signal magnitude of non-specific controls (RAF Abs to TNFa and RAF diclofenac) as compared with the placebo ranged from 4.8% to 6.0%. Hence, RAF Abs to IFNg are capable of specifically interfering with Ag-Abs binding (Figure 2).

% = Δf ((RAF Abs to IFNg) − Δf (control)) * 100%/Δf (control), * means *p* < 0.0001

The detection of the RAF of Abs was a challenge while developing such a class of drugs. The appropriate method for determination of RAF of Abs activity should be based on their mechanism of action and be sensitive enough to detect small changes in interaction of the molecules. As we stated before, the RAF of Abs exert the direct effect on conformational parameters of the biological target molecules which is a probable trigger mechanism resulting in sensitization of the biological targets and modification of the parameters of ligand-receptor or antigen-antibody interactions [27]. The PI approach seemed to be very convenient for solving this kind of task. Our results confirmed this suggestion and showed that PI devices are applicable for the detection of RAF Abs to IFNg samples (formulated as solutions, lactose powders or tablets) by examining their effects on the interaction between IFNg and specific Abs.

During method development, the preliminary testing stage was included to perform an analysis of experimental conditions which involved the selection of the optimal coating for the electrode surface, detection conditions and the proper chaotropic agent for electrode regeneration. In addition, the analytical signals obtained during the experiments had low relative deviations, both for short-term measurements and for tests repeated on different days by different operators, suggesting high repeatability and intermediate precision of the analytical procedure. The piezoelectric sensor technique was shown to have high specificity, allowing the specific identification of RAF Abs to IFNg in samples in comparison with controls.

## 4. Conclusions

Thus, the developed assay could be used both for regular detection of IFNg-antibody to IFNg interactions and for identification of RAF Abs to IFNg in solutions, lactose powders or tablet samples in comparison with controls. The established technique is reliable, reproducible and easy to perform. Obtained data support findings received during ELISA method development [40] showing the ability of RAF Abs to IFNg to modify IFNg affinity to specific Abs to IFNg and encourage our assumption about the RAF Abs’ effect on the spatial Ag-Abs interactions. Nevertheless, further studies into this field are contemplated.

## Supplementary Materials

The following are available online at http://www.mdpi.com/1424-8220/16/1/96/s1, Table S1: Studies of the affinity of biolayer coating obtained using various preparation methods for different Abs concentrations. Table S2: Effects of different chaotropic agents on the number of measurement runs N (MU used for receptor layer coating). Table S3: Repeatability evaluation of net signal measurements for target samples *vs.* reference solution full data. Table S4: Intermediate precision evaluation of net signal measurements for target samples *vs.* reference solution (*n* = 150, *p* = 0.95) full data.
