# Supplementary material for: Use of Piezoelectric Immunosensors for Detection of Interferon-Gamma Interaction with Specific Antibodies in the Presence of Released-Active Forms of Antibodies to Interferon-Gamma
Source: Sensors (Basel). 2016 Jan 20;16(1):96. doi: 10.3390/s16010096 (PMC4732129; doi:10.3390/s16010096)
Supplement: Supplementary file 1 [file sensors-16-00096-s001.pdf]

Supplementary Material: Use of Piezoelectric Immunosensors for Detection of Interferon-Gamma Interaction with Specific Antibodies in the Presence of Released-Active Forms of Antibodies to Interferon-Gamma

Elena Don, Olga Farafonova, Suzanna Pokhil, Darya Barykina [1], Marina Nikiforova, Darya Shulga, Alena Borshcheva, Sergey Tarasov, Tatyana Ermolaeva and Oleg Epstein

Table S1. Studies of the affinity of biolayer coating obtained using various preparation methods for different Abs concentrations.

Parameter	Method 1	Method 2	Method 3	Method 4			Method 5	
				MU + AMT	MU + OMBP	MU + MPT	GA	Calix[4]rene
undiluted Abs to IFNg								
f_1	9310307	9725769	9487605	9858925	10005262	9985632	9535614	
f (mean, *n* = 5) ± SD	9309630 ± 192.5	9723476 ± 140400.8	9485924 ± 124989.8	9858307 ± 225.1			9534996 ± 310574	
Δf	676.6	2293.4	1681.2	618.4			617.6	–
M	832.218	2820.882	2067.876	760.632	–	Sensor overloading	759.648	
	155.618	527.482	386.676	142.232			142.048	
N	2	1	3	9			5	–
A	10149	34401	25218	9276			9264	
Abs to IFNg diluted two-fold								
f_1	9079825	9167912	9238957		9985126	10051563	8771563	
f (mean, *n* = 5) ± SD	9079217 ± 130567	9166859 ± 311177.5	9238592 ± 412577.2		9985034 ± 23192	10031111 ± 313035	8770837 ± 71454	
Δf	607.8	1053.4	365.4		91.6	20451.6	726.4	
M	747.594	1295.682	449.442	–	112.668	25155.47	893.472	–
H	139.794	242.282	84.042		21.068	4703.868	167.072	
N	6	7	20		5	4	16	
A	9117	15801	5481		1374	306774	10896	
Abs to IFNg diluted four-fold								
f_1	9228963	9013968	9400487	9850064			8902896	9888215
f (mean, *n* = 5) ± SD	9228734 ± 45222	9013371 ± 405605	9400239 ± 321144	9849175 ± 156917			8902760 ± 215630	9888118 ± 83486
Δf	228.8	596.8	248	888.6			135.8	97.2
M	281.424	734.064	305.04	1092.978	–	–	167.034	119.556
H	52.624	137.264	57.04	204.378			31.234	22.356
N	12	5	21	21			18	11
A	3432	8952	3720	13329			2037	1458

Annotation for Supplementary Table S1. Δf—oscillation frequency of the sensor following Abs-IFNg binding (Hz); f (mean)—mean oscillation frequency from 5 replicate tests (Hz); f_1—oscillation frequency of the sensor before contact with IFNg (Hz); m—mass of bioreceptor layer (ng) prepared using methods 1–5; h—bioreceptor layer thickness (nm); N—number of measurement runs where the analytical signal was not decreased by more than 5%; A—concentration sensitivity (A. Hz·mL·ng^{-1}) reflecting variations in oscillation frequency of the immunosensor upon contact with IFNg.

Table S2. Effects of different chaotropic agents on the number of measurement runs N (MU used for receptor layer coating).

Chaotropic Agent	*n*
Potassium rhodanide	21
Hydroxylamine hydrochloride	20–21
Urea	8
Phenol	6
Diethylamine hydrochloride	20–21
Magnesium chloride	15
Bidistilled water	–

Table S3. Repeatability evaluation of net signal measurements for target samples *vs.* reference solution full data.

Analytical Signal of Sensor	Number of Experiment	Sample *vs.* IFNg Ratio					
		1:1	1:2	1:4	1:6	2:1	4:1
RAF of Abs to IFNg (liquid samples)							
	1	252	308	405	441	189	125
	2	248	305	402	425	196	126
	3	241	307	395	439	191	120
Δf	4	260	303	408	436	205	131
	5	252	311	405	452	192	118
	average ± δ	251 ± 9	307 ± 4	403 ± 6	439 ± 12	195 ± 8	124 ± 6
	s	252	308	405	441	189	125
Placebo (liquid samples)							
	1	295	363	412	445	223	167
	2	289	358	425	451	225	168
	3	287	367	421	448	218	165
Δf	4	298	364	417	462	219	162
	5	292	359	419	448	214	161
	average ± δ	292 ± 6	362 ± 5	419 ± 6	451 ± 8	220 ± 5	165 ± 4
	s	4	4	5	7	4	3
RAF of Abs to IFNg (tablets)							
	1	245	295	389	436	215	112
	2	251	298	385	425	218	109
	3	256	289	392	419	209	118
Δf	4	239	291	378	426	225	121
	5	254	296	396	429	218	108
	average ± δ	249 ± 9	294 ± 5	388 ± 9	427 ± 8	217 ± 7	114 ± 7
	s	7	4	7	6	6	6
Placebo (tablets)							
	1	302	401	426	458	230	189
	2	306	412	435	479	235	196
	3	315	413	431	465	231	193
Δf	4	309	405	429	481	227	187
	5	307	408	418	459	209	185
	average ± δ	308 ± 6	408 ± 6	428 ± 8	468 ± 14	226 ± 13	190 ± 6
	s	5	5	6	11	10	4

Table S3. *Cont.*

Analytical Signal of Sensor	Number of Experiment	Sample *vs.* IFNg Ratio					
		**1:1**	**1:2**	**1:4**	**1:6**	**2:1**	**4:1**
RAF of Abs to IFNg (lactose powders)							
	1	215	286	379	445	178	95
	2	202	276	382	439	165	109
	3	218	282	359	421	159	112
Δf	4	221	286	387	452	176	125
	5	204	274	386	461	156	106
	average ± δ	212 ± 11	281 ± 7	379 ± 14	444 ± 19	167 ± 12	109 ± 13
	s	9	6	11	15	10	11
Placebo (lactose powders)							
	1	287	360	402	456	205	142
	2	279	351	425	459	211	153
	3	285	362	409	438	198	149
Δf	4	290	378	387	432	204	143
	5	286	359	401	459	209	148
	average ± δ	285 ± 5	362 ± 12	405 ± 17	449 ± 16	205 ± 6	147 ± 6
	s	4	10	14	13	5	5

Table S4. Intermediate precision evaluation of net signal measurements for target samples *vs.* reference solution (n = 150, p = 0.95) full data.

**Day 1.**				
**Analytical Signal of Sensor**	**Number of Experiment**	**Operator 1**	**Operator 2**	**Operator 3**
RAF of Abs to IFNg (liquid samples)				
	1	152	145	161
	2	154	147	144
	3	145	154	152
Δf	4	149	153	162
	5	141	148	155
	average ± δ	148 ± 7	149 ± 5	155 ± 9
	s	5	4	7
Placebo (liquid samples)				
	1	201	198	206
	2	194	215	200
	3	204	214	196
Δf	4	213	196	197
	5	205	208	205
	average ± δ	203 ± 9	206 ± 11	201 ± 6
	s	7	9	5
RAF of Abs to IFNg (tablets)				
	1	122	104	102
	2	118	109	108
	3	116	115	111
Δf	4	101	111	115
	5	107	121	104
	average ± δ	113 ± 11	112 ± 8	108 ± 7
	s	9	6	5

Table S4. *Cont.*

Day 1.				
**Analytical Signal of Sensor**	**Number of Experiment**	**Operator 1**	**Operator 2**	**Operator 3**
Placebo (tablets)				
Δf	1	169	156	150
	2	174	154	159
	3	181	157	165
	4	170	169	168
	5	180	162	171
	average ± δ	175 ± 7	160 ± 7	163 ± 10
	s	6	6	8
RAF of Abs to IFNg (lactose powders)				
Δf	1	108	114	113
	2	104	106	110
	3	112	104	103
	4	101	109	114
	5	109	111	105
	average ± δ	107 ± 5	109 ± 5	109 ± 6
	s	4	4	5
Placebo (lactose powders)				
Δf	1	167	161	163
	2	162	159	167
	3	163	171	170
	4	174	165	164
	5	176	163	160
	average ± δ	168 ± 8	164 ± 6	165 ± 5
	s	6	5	4
**Day 2.**				
**Analytical Signal of Sensor**	**Number of Experiment**	**Operator 1**	**Operator 2**	**Operator 3**
RAF Abs to IFNg (liquid samples)				
Δf	1	149	151	157
	2	143	156	159
	3	152	157	163
	4	153	149	167
	5	154	150	155
	average ± δ	150 ± 6	153 ± 5	160 ± 6
	s	4	4	5
Placebo (liquid samples)				
Δf	1	203	193	200
	2	207	197	197
	3	198	198	193
	4	205	190	203
	5	206	197	205
	average ± δ	204 ± 4	195 ± 4	200 ± 6
	s	4	3	5

Table S4. *Cont.*

Analytical Signal of Sensor	Number of Experiment	Operator 1	Operator 2	Operator 3
**Day 2.**				
**Analytical Signal of Sensor**	**Number of Experiment**	**Operator 1**	**Operator 2**	**Operator 3**
RAF of Abs to IFNg (tablets)				
Δf	1	125	109	109
	2	114	115	115
	3	109	123	123
	4	118	117	117
	5	112	120	120
	average ± δ	116 ± 8	117 ± 7	117 ± 7
	s	6	5	5
Placebo (tablets)				
Δf	1	172	165	165
	2	176	158	168
	3	184	169	151
	4	165	152	158
	5	178	160	164
	average ± δ	175 ± 9	161 ± 8	161 ± 8
	s	7	7	7
RAF of Abs to IFNg (lactose powders)				
Δf	1	108	110	115
	2	104	115	108
	3	112	108	119
	4	101	107	121
	5	109	114	108
	average ± δ	107 ± 5	111 ± 4	114 ± 8
	s	4	4	6
Placebo (lactose powders)				
Δf	1	169	169	156
	2	164	172	152
	3	162	160	165
	4	170	157	161
	5	165	155	154
	average ± δ	166 ± 4	163 ± 9	158 ± 7
	s	3	8	5
**Day 3.**				
**Analytical Signal of Sensor**	**Number of Experiment**	**Operator 1**	**Operator 2**	**Operator 3**
RAF of Abs to IFNg (liquid samples)				
Δf	1	147	144	143
	2	154	154	145
	3	149	147	152
	4	154	155	150
	5	156	142	143
	average ± δ	152 ± 5	148 ± 7	147 ± 5
	s	4	6	4

Table S4. *Cont.*

Day 3.				
**Analytical Signal of Sensor**	**Number of Experiment**	**Operator 1**	**Operator 2**	**Operator 3**
Placebo (liquid samples)				
Δf	1	207	193	204
	2	200	199	201
	3	191	210	191
	4	200	199	206
	5	192	207	197
	average ± δ	198 ± 8	202 ± 8	200 ± 7
	s	7	7	6
RAF of Abs to IFNg (tablets)				
Δf	1	121	120	124
	2	117	122	114
	3	109	121	114
	4	115	109	118
	5	114	119	111
	average ± δ	115 ± 5	118 ± 7	116 ± 6
	s	4	5	5
Placebo (tablets)				
Δf	1	173	175	171
	2	184	176	183
	3	182	169	183
	4	176	169	176
	5	180	181	176
	average ± δ	179 ± 6	174 ± 6	178 ± 6
	s	4	5	5
RAF of Abs to IFNg (lactose powders)				
Δf	1	118	107	100
	2	103	117	106
	3	105	118	100
	4	119	114	118
	5	109	108	120
	average ± δ	111 ± 9	113 ± 6	109 ± 12
	s	7	5	10
Placebo (lactose powders)				
Δf	1	173	155	159
	2	172	169	169
	3	169	170	155
	4	165	168	163
	5	175	157	157
	average ± δ	171 ± 5	164 ± 9	161 ± 7
	s	4	7	6

Table S4. *Cont.*

Day 4.				
**Analytical Signal of Sensor**	**Number of Experiment**	**Operator 1**	**Operator 2**	**Operator 3**
RAF of Abs to IFNg (liquid samples)				
	1	153	144	144
	2	145	153	152
	3	149	155	157
Δf	4	145	144	146
	5	149	153	145
	average ± δ	148 ± 4	150 ± 7	149 ± 7
	s	3	5	6
Placebo (liquid samples)				
	1	201	196	203
	2	200	198	193
	3	202	191	203
Δf	4	196	202	202
	5	194	208	195
	average ± δ	199 ± 4	199 ± 8	199 ± 6
	s	3	6	5
RAF of Abs to IFNg (tablets)				
	1	113	109	131
	2	134	107	103
	3	102	131	123
Δf	4	111	109	101
	5	124	111	111
	average ± δ	117 ± 2	113 ± 1	114 ± 2
	s	12	10	13
Placebo (tablets)				
	1	168	172	192
	2	166	174	186
	3	190	180	186
Δf	4	175	188	169
	5	181	174	173
	average ± δ	176 ± 1	178 ± 8	181 ± 1
	s	10	7	10
RAF of Abs to IFNg (lactose powders)				
	1	118	115	100
	2	103	111	106
	3	105	112	100
Δf	4	119	112	118
	5	109	115	120
	average ± δ	111 ± 7	113 ± 2	109 ± 1
	s	14	4	10

Table S4. *Cont.*

**Day 4.**				
**Analytical Signal of Sensor**	**Number of Experiment**	**Operator 1**	**Operator 2**	**Operator 3**
Placebo (lactose powders)				
	1	152	175	180
	2	176	167	156
	3	175	157	164
Δf	4	173	171	165
	5	158	176	175
	average ± δ	167 ± 1	169 ± 1	168 ± 1
	s	11	8	10

**Day 5.**				
**Analytical Signal of Sensor**	**Number of Experiment**	**Operator 1**	**Operator 2**	**Operator 3**
RAF of Abs to IFNg (liquid samples)				
	1	157	143	148
	2	151	153	156
	3	151	153	154
Δf	4	158	150	147
	5	151	149	142
	average ± δ	154 ± 4	150 ± 5	149 ± 7
	s	4	4	6
Placebo (liquid samples)				
	1	196	206	190
	2	202	200	195
	3	201	196	202
Δf	4	206	192	207
	5	196	203	209
	average ± δ	200 ± 5	199 ± 7	201 ± 10
	s	4	6	8
RAF of Abs to IFNg (tablets)				
	1	118	119	110
	2	109	115	122
	3	113	106	115
Δf	4	104	110	111
	5	120	112	109
	average ± δ	113 ± 8	112 ± 6	113 ± 7
	s	7	5	5
Placebo (tablets)				
	1	185	170	177
	2	182	177	179
	3	175	169	183
Δf	4	176	182	174
	5	184	173	169
	average ± δ	180 ± 6	174 ± 7	176 ± 7
	s	5	5	5

Table S4. *Cont.*

**Day 5.**				
**Analytical Signal of Sensor**	**Number of Experiment**	**Operator 1**	**Operator 2**	**Operator 3**
RAF of Abs to IFNg (lactose powders)				
	1	103	118	113
	2	107	107	109
	3	111	119	109
Δf	4	117	118	109
	5	112	116	116
	average ± δ	110 ± 7	116 ± 6	111 ± 4
	s	5	5	3
Placebo (lactose powders)				
	1	171	176	176
	2	177	173	164
	3	165	165	157
Δf	4	173	166	172
	5	170	162	175
	average ± δ	171 ± 5	168 ± 7	169 ± 10
	s	4	6	8
**Day 6.**				
**Analytical Signal of Sensor**	**Number of Experiment**	**Operator 1**	**Operator 2**	**Operator 3**
RAF of Abs to IFNg (liquid samples)				
	1	161	145	151
	2	149	147	153
	3	152	162	161
Δf	4	161	162	146
	5	148	161	154
	average ± δ	154 ± 8	155 ± 1	153 ± 7
	s	6	9	5
Placebo (liquid samples)				
	1	212	200	193
	2	201	204	206
	3	203	197	204
Δf	4	196	206	198
	5	202	204	205
	average ± δ	203 ± 7	202 ± 5	201 ± 7
	s	6	4	6
RAF of Abs to IFNg (tablets)				
	1	118	113	106
	2	111	107	109
	3	121	119	112
Δf	4	115	110	120
	5	110	113	124
	average ± δ	115 ± 6	112 ± 6	114 ± 9
	s	5	4	8

Table S4. *Cont.*

Day 6.				
**Analytical Signal of Sensor**	**Number of Experiment**	**Operator 1**	**Operator 2**	**Operator 3**
Placebo (tablets)				
	1	179	184	179
	2	185	177	178
	3	169	179	169
Δf	4	176	178	184
	5	176	170	172
	average ± δ	177 ± 7	178 ± 6	176 ± 7
	s	6	5	6
RAF of Abs to IFNg (lactose powders)				
	1	118	108	104
	2	118	117	122
	3	123	118	104
Δf	4	105	113	121
	5	121	103	116
	average ± δ	117 ± 9	112 ± 8	113 ± 1
	s	7	6	9
Placebo (lactose powders)				
	1	175	179	161
	2	169	174	171
	3	172	164	178
Δf	4	168	165	171
	5	177	164	178
	average ± δ	172 ± 5	169 ± 9	172 ± 9
	s	4	7	7
**Day 7.**				
**Analytical Signal of Sensor**	**Number of Experiment**	**Operator 1**	**Operator 2**	**Operator 3**
RAF of Abs to IFNg (liquid samples)				
	1	166	153	149
	2	168	150	158
	3	158	166	169
Δf	4	152	165	166
	5	152	151	157
	average ± δ	159 ± 9	157 ± 1	160 ± 1
	s	8	8	8
Placebo (liquid samples)				
	1	207	201	211
	2	195	204	215
	3	209	201	218
Δf	4	214	199	198
	5	202	198	214
	average ± δ	205 ± 9	201 ± 3	211 ± 1
	s	7	2	8

Table 4. *Cont.*

**Day 7.**				
**Analytical Signal of Sensor**	**Number of Experiment**	**Operator 1**	**Operator 2**	**Operator 3**
RAF of Abs to IFNg (tablets)				
	1	107	109	114
	2	119	124	124
	3	114	124	105
Δf	4	120	126	119
	5	106	114	121
	average ± δ	113 ± 8	119 ± 9	117 ± 9
	s	7	7	7
Placebo (tablets)				
	1	184	169	168
	2	179	167	188
	3	180	165	174
Δf	4	174	165	182
	5	167	188	169
	average ± δ	177 ± 8	171 ± 1	176 ± 1
	s	7	10	9
RAF of Abs to IFNg (lactose powders)				
	1	121	102	117
	2	120	102	103
	3	111	106	102
Δf	4	120	119	106
	5	101	114	110
	average ± δ	115 ± 9	109 ± 8	108 ± 8
	s	11	9	8
Placebo (lactose powders)				
	1	178	182	166
	2	166	169	175
	3	180	161	167
Δf	4	169	167	160
	5	173	180	181
	average ± δ	173 ± 7	172 ± 1	170 ± 1
	s	6	9	8
**Day 8.**				
**Analytical Signal of Sensor**	**Number of Experiment**	**Operator 1**	**Operator 2**	**Operator 3**
RAF of Abs to IFNg (liquid samples)				
	1	172	158	157
	2	157	157	168
	3	171	149	165
Δf	4	166	163	156
	5	158	169	156
	average ± δ	165 ± 9	159 ± 9	160 ± 7
	s	7	7	6

Table S4. *Cont.*

Analytical Signal of Sensor	Number of Experiment	Operator 1	Operator 2	Operator 3
**Day 8.**				
Placebo (liquid samples)				
	1	201	191	211
	2	195	207	212
	3	203	202	214
Δf	4	202	196	203
	5	208	215	210
	average ± δ	202 ± 6	202 ± 1	210 ± 5
	s	5	9	4
RAF of Abs to IFNg (tablets)				
	1	114	123	118
	2	118	115	107
	3	122	118	113
Δf	4	123	113	104
	5	110	106	124
	average ± δ	117 ± 7	115 ± 8	113 ± 1
	s	5	6	8
Placebo (tablets)				
	1	181	173	180
	2	169	179	174
	3	171	178	176
Δf	4	172	169	182
	5	176	176	178
	average ± δ	174 ± 6	175 ± 5	178 ± 4
	s	5	4	3
RAF of Abs to IFNg (lactose powders)				
	1	106	104	107
	2	116	116	105
	3	101	118	119
Δf	4	103	102	103
	5	120	116	112
	average ± δ	109 ± 1	111 ± 9	109 ± 8
	s	8	8	6
Placebo (lactose powders)				
	1	173	178	171
	2	179	177	164
	3	175	180	180
Δf	4	166	179	170
	5	163	178	174
	average ± δ	171 ± 8	178 ± 1	172 ± 7
	s	7	1	6

Table S4. *Cont.*

Day 9.				
**Analytical Signal of Sensor**	**Number of Experiment**	**Operator 1**	**Operator 2**	**Operator 3**
RAF of Abs to IFNg (liquid samples)				
	1	167	153	173
	2	170	154	153
	3	158	175	161
Δf	4	165	166	152
	5	160	166	154
	average ± δ	164 ± 6	163 ± 11	159 ± 11
	s	5	9	9
Placebo (liquid samples)				
	1	190	199	200
	2	203	207	192
	3	203	188	193
Δf	4	205	199	210
	5	209	194	193
	average ± δ	202 ± 9	197 ± 9	198 ± 9
	s	7	7	8
RAF of Abs to IFNg (tablets)				
	1	124	121	129
	2	111	124	121
	3	116	124	115
Δf	4	114	115	129
	5	107	121	108
	average ± δ	114 ± 8	121 ± 5	120 ± 1
	s	6	4	9
Placebo (tablets)				
	1	163	170	171
	2	171	180	176
	3	173	162	179
Δf	4	174	174	182
	5	182	162	178
	average ± δ	173 ± 8	170 ± 1	177 ± 5
	s	7	8	4
RAF of Abs to IFNg (lactose powders)				
	1	104	109	113
	2	119	123	106
	3	111	113	127
Δf	4	114	125	107
	5	122	107	115
	average ± δ	114 ± 9	115 ± 1	114 ± 1
	s	7	8	8

Table S4. *Cont.*

**Day 9.**				
**Analytical Signal of Sensor**	**Number of Experiment**	**Operator 1**	**Operator 2**	**Operator 3**
Placebo (lactose powders)				
Δf	1	172	172	175
	2	179	166	170
	3	186	170	165
	4	186	186	185
	5	172	179	165
	average ± δ	179 ± 9	175 ± 1	172 ± 1
	s	7	8	8

**Day 10.**				
**Analytical Signal of Sensor**	**Number of Experiment**	**Operator 1**	**Operator 2**	**Operator 3**
RAF of Abs to IFNg (liquid samples)				
Δf	1	158	165	160
	2	172	160	163
	3	162	166	155
	4	166	173	161
	5	160	160	164
	average ± δ	164 ± 7	165 ± 7	161 ± 4
	s	6	5	4
Placebo (liquid samples)				
Δf	1	204	210	195
	2	189	207	200
	3	195	212	196
	4	202	197	209
	5	208	199	197
	average ± δ	200 ± 9	205 ± 8	199 ± 7
	s	8	7	6
RAF of Abs to IFNg (tablets)				
Δf	1	115	129	107
	2	115	113	130
	3	124	108	109
	4	112	126	111
	5	111	120	114
	average ± δ	115 ± 6	119 ± 1	114 ± 1
	s	5	9	9
Placebo (tablets)				
Δf	1	167	182	168
	2	169	183	187
	3	187	166	166
	4	171	167	181
	5	166	171	182
	average ± δ	172 ± 1	174 ± 1	177 ± 1
	s	9	8	9

Table S4. *Cont.*

**Day 10.**				
**Analytical Signal of Sensor**	**Number of Experiment**	**Operator 1**	**Operator 2**	**Operator 3**
RAF of Abs to IFNg (lactose powders)				
	1	120	110	111
	2	117	116	119
	3	102	103	122
Δf	4	113	102	116
	5	112	115	115
	average ± δ	113 ± 8	109 ± 8	117 ± 5
	s	7	7	4
Placebo (lactose powders)				
	1	167	177	169
	2	164	171	166
	3	183	171	176
Δf	4	186	181	186
	5	175	178	168
	average ± δ	175 ± 12	176 ± 6	173 ± 1
	s	10	4	8
